# Supplementary material for: DeepRaccess: high-speed RNA accessibility prediction using deep learning
Source: Front Bioinform. 2023 Oct 10;3:1275787. doi: 10.3389/fbinf.2023.1275787 (PMC10597636; doi:10.3389/fbinf.2023.1275787)
Supplement: Supplementary file 1 [file DataSheet1.PDF]

Supplementary Materials

Supplementary Figures

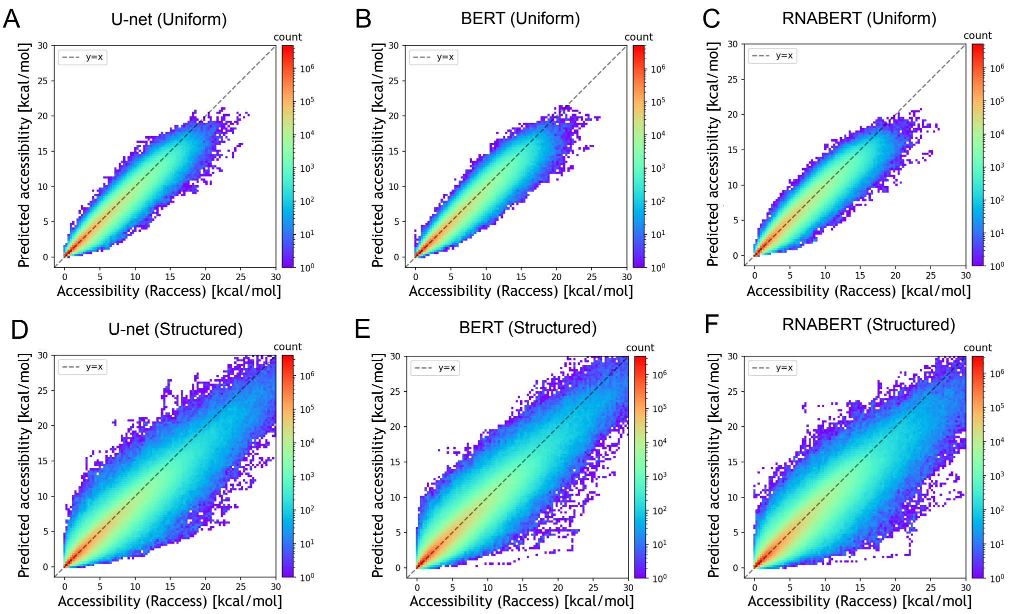

Fig. S1 Prediction performances for the simulation datasets. Accuracy of the uniform RNA dataset for (A) the U-net model, (B) the BERT model, and (C) the RNABERT model. Accuracy of the structured RNA dataset for (D) the U-net model, (E) the BERT model, and (F) the RNABERT model. The x and y axes represent the accessibility calculated by Raccess and predicted accessibility, respectively. The color bar representing the counts is displayed using a log scale. The prediction performances of the simulation datasets for FCN was provided as Fig. 2.

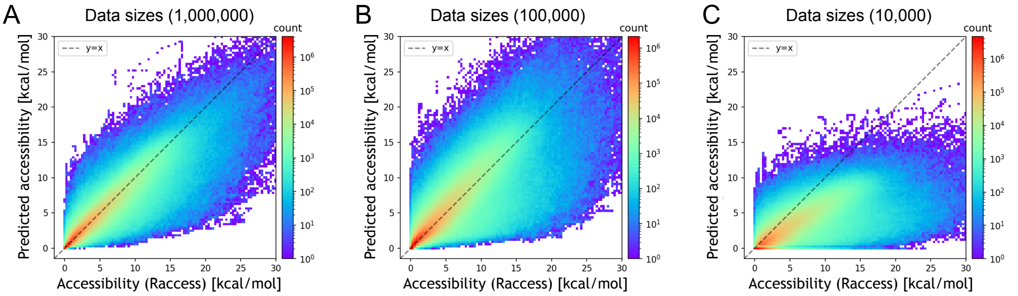

Fig. S2 Influence of the training data sizes on the prediction performances. The data sizes were (A) 1,000,000, (B)100,000, and (C) 10,000. The x and y axes represent the accessibility calculated by Raccess and predicted accessibility, respectively. The color bar representing the counts is displayed using a log scale.

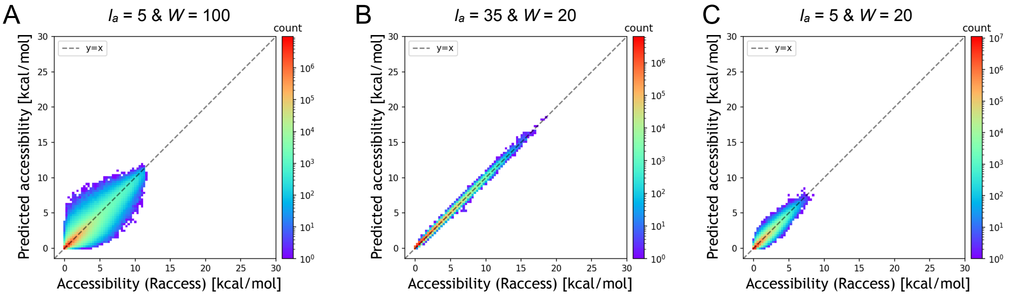

Fig. S3 Influence of the parameters l_a and W on the prediction performances. (A) $l_a = 5$ and $W = 100$. (B) $l_a = 35$ and $W = 20$. (C) $l_a = 5$ and $W = 20$. The x and y axes represent the accessibility calculated by Raccess and predicted accessibility, respectively. The color bar representing the counts is displayed using a log scale.

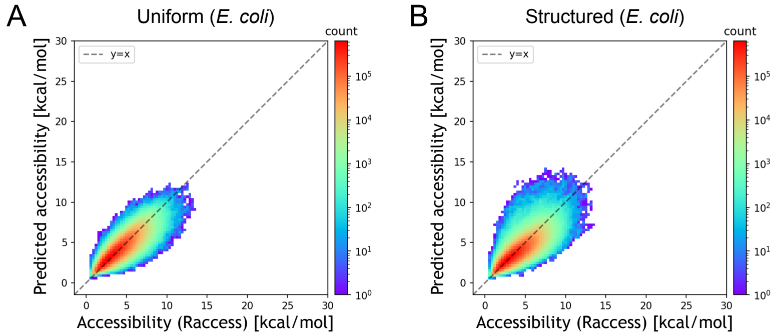

Fig. S4 Prediction accuracy for the *E.coli* synthetic RNA datasets of the predictive model trained on (A) the uniform RNA dataset and (B) the structured RNA dataset. The x and y axes represent the accessibility calculated by Raccess and predicted accessibility, respectively. The color bar representing the counts is displayed using a log scale.

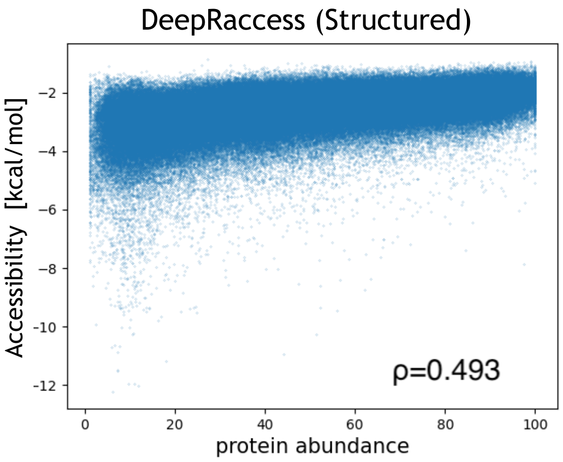

Fig. S5 Correlation between the protein abundance and the accessibility calculated by DeepRaccess trained with the structured RNA dataset. The protein abundance was measured by fluorescence-activated cell sorting and was normalized so that the minimum value was 1 and the maximum value was 100. The x- and y-axis represent the protein abundance and the accessibility, respectively.

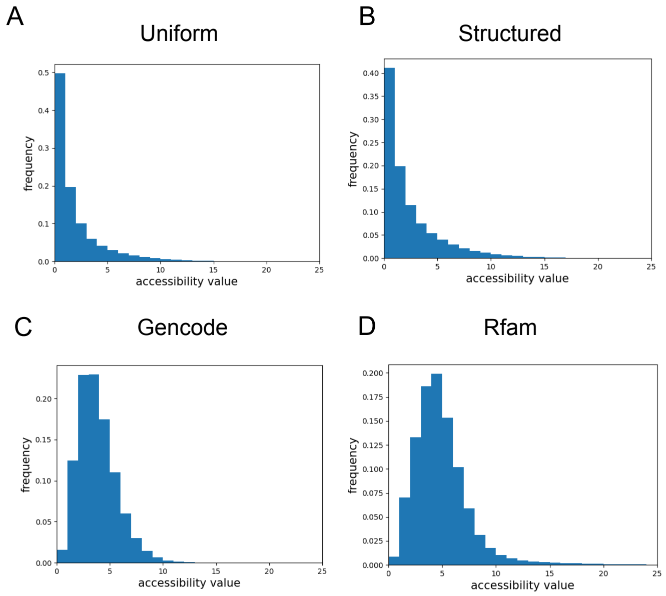

Fig. S6 Distributions of accessibility for (A) the uniform RNA dataset, (B) the structured RNA dataset, (C) the Gencode and (D) the Rfam datasets. The x- and y-axis represent the accessibility and the frequency, respectively.

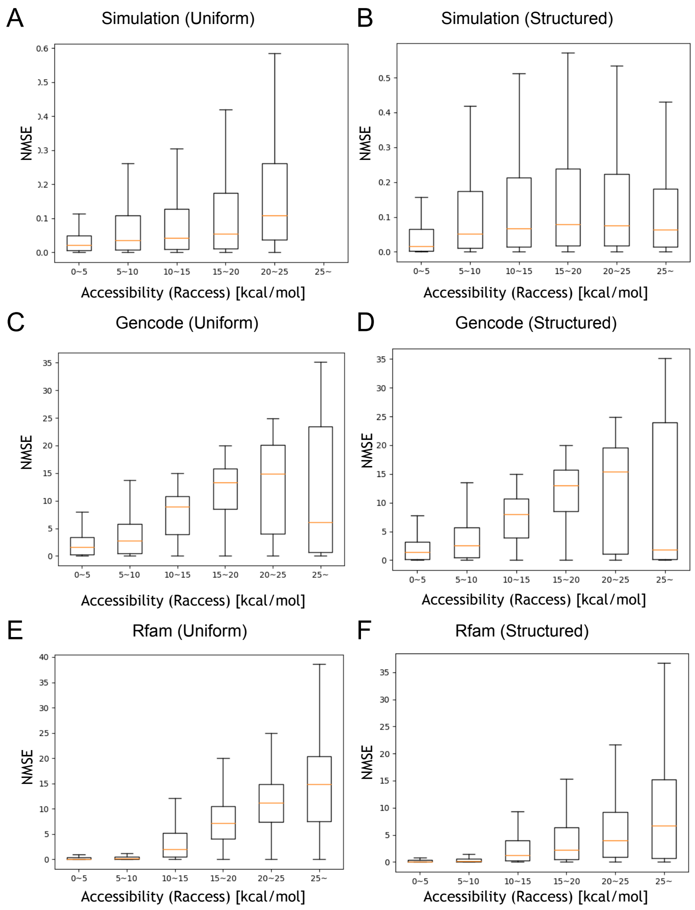

Fig. S7 Correlation between the accessibility and the NMSE for (A) the uniform RNA dataset, (B) the structured RNA dataset, (C-D) the Gencode dataset and (E-F) the Rfam datasets. DeepRaccess was trained with (A, C, E) the uniform RNA dataset and (B, D, F) the structured RNA dataset. We excluded the outlier from the analysis. The x- and y-axes represent the accessibility calculated by Raccess and the NMSE, respectively.

Supplementary Tables

Table S1 Architecture of the FCN model

module	parameter	shape	notes
input		(440)	
embedding	dim = 120	(120, 440)	
1D CNN BN Mish	chnnel=120, kernel=9, padding=4	(120, 440)	
1D CNN BN ReLU	chnnel=120, kernel=5, dilation=3, padding=6	(120, 440)	× 40
1D CNN BN Mish	chnnel=120, kernel=9, padding=4	(120, 440)	
1D CNN BN ReLU	chnnel=1, kernel=9, padding=4	(1, 440)	
output		(440)	

Table S2 Architecture of the U-net model

module	parameter	shape	notes
input		(440)	
embedding	dim = 120	(120, 440)	
1D CNN BN ReLU	chnnel=240, kernel=5, padding=2, stride = 2	(240, 220)	save as X
1D CNN BN ReLU	chnnel=360, kernel=5, padding=2, stride = 2	(360, 110)	save as Y
1D CNN BN ReLU	chnnel=480, kernel=5, padding=2, stride = 2	(480, 55)	save as Z
1D CNN BN ReLU	chnnel=480, kernel=5, dilation = 3, padding=6	(480, 55)	× 35
1D transposedCNN BN ReLU	chnnel=360, kernel=5, padding=2, stride = 2	(360, 110)	+Z
1D transposedCNN BN ReLU	chnnel=240, kernel=5, padding=2, stride = 2	(240, 220)	+Y
1D transposedCNN BN ReLU	chnnel=120, kernel=5, padding=2, stride = 2	(120, 440)	+X
1D CNN BN Mish	chnnel=120, kernel=9, padding=4	(120, 440)	
1D CNN BN ReLU	chnnel=1, kernel=9, padding=4	(1, 440)	
output		(440)	

Table S3 Architecture of the BERT model

module	parameter	shape	notes
input		(440)	
BERT	attention heads=12, hidden layers=6	(120, 440)	
1D CNN BN Mish	chnnel=120, kernel=9, padding=4	(120, 440)	
1D CNN BN ReLU	chnnel=1, kernel=9, padding=4	(1, 440)	
output		(440)	

Table S4 Architecture of the RNABERT model

module	parameter	shape	notes
input		(440)	
BERT	attention heads=12，hidden layers=6	(120，440)	pretrained model
1D CNN BN Mish	chnnel=120，kernel=9，padding=4	(120，440)	
1D CNN BN ReLU	chnnel=1，kernel=9，padding=4	(1，440)	
output		(440)	

Table S5 Influence of the training data sizes on the prediction performances

Data sizes	NMSE	Spearmans' ρ
10,000	1.6998	0.8601
100,000	0.6762	0.9449
1,000,000	0.3892	0.9688
10,000,000	0.1148	0.9876

Table S6 Influence of the parameters l_a and W on the prediction performances

Parameters	NMSE	Spearmans' ρ
$l_a = 5, W = 20$	0.0406	0.9744
$l_a = 5, W = 100$	0.1396	0.9706
$l_a = 35, W = 20$	0.0050	0.9995
$l_a = 35, W = 100$	0.1148	0.9876
